# Address of Dr. E. Parmly to the Graduating Class of the Baltimore College of Dental Surgery

**Published:** 1849-04

**Authors:** 


### ADDRESS OF DR. E. PARMLY TO THE GRADUATING CLASS OF THE BALTIMORE COLLEGE OF DENTAL SURGERY.

Our attention has been drawn, by our friend, Dr. E. Parmly, to a typographical error which occurs in his address to the late graduating class of the Baltimore School of Dental Surgery. We can assure the Doctor that the institution of which he so kindly takes notice, located in Cincinnati, would be glad to receive a share of his "*paternal regard.*" The address contains so much valuable thought and advice, that we should like to transfer it to the Register entire. We hope we shall, however, be excused for a lengthy extract, which contains the error alluded to, and which we correct, making it read *fraternal* instead of *paternal*, as published in the American Journal and Library of Dental Science.—[Cin. Ed.

There are perhaps an equal number of those who underrate as those who overrate their abilities, and both of these classes lose the benefit of the knowledge and experience of their seniors. The first, from want of confidence in themselves, or from diffidence, are afraid to approach those who have been long in practice. The second class never approach them, because they believe they have nothing to learn; their self-pride is such that they would feel humbled in either asking or taking advice. Beware, my young friends, of arrogance and pride—they ruin the success and reputation of more dentists in the estimation of the public, and confer fewer benefits, than any thing I know of, except imposture and laziness. The confidence and name gained by generous acts will be of much higher value to you than money gained by mean ones. Meanness in underrating and underpricing your contemporaries should be no part of a professional man's character. It degrades instead of elevates any and every one who will do it. You can succeed, if you will, without such unmanly and ignoble artifices. Perseverance and industry, with the knowledge you now possess, never has failed and never will fail of accomplishing all you desire.

I read, a short time ago, from a document written by a dentist of some twenty years' experience, that "dentistry is an art acquired by observation and practice—practice is nearly every thing—theory very little." There may be others who would express the same opinion; but when they do so, they show themselves wholly unacquainted with, and ignorant of, the best and true sources of professional knowledge, and an entire indifference to the value of correct theory, from which must result all correct practice. This remark should be exactly reversed with the young practitioner: theory is nearly every thing—practice, or the mere manipulation, is comparatively very little, as you must all have experienced that it is very easy to learn to do a thing after you have been carefully and thoroughly instructed in the best way or mode of doing it. I affirm, and do so without the fear of contradiction, that the true surgical and mechanical principles or science of our art, your acquaintance with, and your ability to perform safely, the delicate and beautiful operations exhibited by graduates of this institution, never was and never will be attained by any one having only the advantage of observation from his own practice. It is now an easy matter to make a lightning rod or construct telegraphic wires; but it required the ablest minds to originate and entertain the theory of electric transmission; and we might, with the same justice and propriety, say, that the making of the wires is nearly every thing, and the mind which conceived, and the theory which established this truly wonderful communication, very little. It is of much greater consequence, my young friends, to yourselves, to your profession, and infinitely greater to your patients, for you to know when and how to perform an operation so as to avoid risk or danger, than merely to be able mechanically to do it well, and, for the want of such knowledge, hazard the health, vitality, or, may be, the entire loss of the teeth upon which you have operated. In this way, there is, at this very period, a greater amount of injury than good done to teeth, and that, too, by the recklessness of persons who submit themselves, without knowledge or proper precaution, to dentists more reckless than themselves.

I trust, in your professional practice, you will never forget that you are graduates of the Baltimore College—the first institution of the kind established in the world; and we have great confidence and strong faith in your efforts to make it known, and regard it not only as first in origin, but first in the character and qualifications of its graduates. And may you become as distinguished in your calling for the superiority of your professional tact and attainments over that of any other system of instruction, and gain as much credit for yourselves, and reflect as much honor on your profession, your instructors, and upon this institution, in contending for truth and correct professional practice against error, imposture, and presumption, as our noble youths, the graduates of the Academy of West Point, have for themselves, their country, and their institution, in their late triumphant conflicts with their enemies on the battle grounds of Mexico. It is with no small degree of pride and pleasure I speak of a sister institution, established on the same basis, in

Cincinnati, Ohio, one of the fields of my early professional efforts. We congratulate its professors and teachers in their laudable designs and philanthropic labors; we commend their enterprise, and most cordially extend to them our fraternal regards and ardent wishes for their abiding welfare and prosperity, and most earnestly do we hope they will not fail to attain the highest object vouchsafed to them by their professional zeal and praiseworthy ambition.

